# Gendered Discrimination Against Immigrants: Experimental Evidence

**DOI:** 10.3389/fsoc.2020.00059

**Published:** 2020-09-03

**Authors:** Johanna Gereke, Max Schaub, Delia Baldassarri

**Affiliations:** ^1^Mannheim Center for European Social Research (MZES), Mannheim, Germany; ^2^Berlin Social Science Center (WZB), Berlin, Germany; ^3^Department of Sociology, New York University, New York, NY, United States

**Keywords:** immigration, ingroup favoritism, pro-social behavior, trust, gendered ethnic discrimination, Germany, behavioral games, experiment

## Abstract

Recent migration from Muslim-majority countries has sparked discussions across Europe about the supposed threat posed by new immigrants. Young men make up the largest share of newly arrived immigrants and this demographic is often perceived to be particularly threatening. In this article, we compare pro-sociality and trust toward immigrants from Muslim-majority countries, focusing on gender differences in treatment. We study these questions using behavioral games that measure strategic (trusting) and non-strategic (pro-social) behavior. Our data comes from measures embedded in a large survey of residents of Germany's eastern regions, where anti-immigrant sentiments are high. We find that Germans are similarly pro-social toward immigrant men and women in non-strategic situations, but are significantly less likely to trust immigrant men (but not women) in strategic encounters. These findings provide evidence that immigrants' gender can be an important factor conditioning the behavior of the majority population, but also caution that (gendered) ethnic discrimination may be situationally dependent. Future research should further examine the exact mechanisms underlying this variation in discriminatory behavior.

## 1. Introduction

According to official estimates, 3.21 million migrants have sought asylum in the European Union during the 2014–2016 refugee crisis, a number unprecedented in recent history (Eurostat, 2017). This sudden inflow in turn generated a heated public debate, aroused anti-immigrant sentiments, and fueled the rise of right-wing populism (Czymara and Schmidt-Catran, 2017; Geiges, 2018; Frey, 2020). Standard accounts explain these developments as reactions to rapid cultural change or demographic threats associated with the arrival of large Muslim populations (Hangartner et al., 2019). However, recent research suggests that an important element underlying exclusionary reactions is that many of these arrivals were young and male (OECD, 2017; Eurostat, 2018). While this demographic often shows high economic potential, it is also frequently viewed as a particularly potent security and cultural threat by some members of the public (Ward, 2019).

Against this backdrop, the present article asks: to what extent are anti-immigrant reactions specifically conditioned on the gender of new arrivals?

To address this question, we present results from behavioral games embedded within a large survey on attitudes toward immigrants and refugees in Germany's eastern region, an area where anti-immigrant sentiments are running high, as manifested in levels of support for the populist right.

We included two standard games: the Dictator Game and the Trust Game. These games are commonly used as workhorse models to assess strategic and non-strategic interactions. To measure discrimination, we randomly varied the gender and ethnicity (German vs. Middle Eastern) of interaction partners. In addition, we also introduced a Split Game in which respondents had to allocate a fixed amount of money between two recipients: one ethnic German and one of immigrant origin. This game captures the common situation in which an individual must choose between natives and immigrants in the allocation of scarce commodities (e.g., access to public housing, educational programs, health services). Discrimination is measured here in terms of deviation from an even 50-50 split between an in-group and out-group member of the same gender.

Overall, different patterns of behavior emerge across our three behavioral games. We do not find any discrimination in the Dictator Game, but record significant anti-immigrant bias in both the Trust and Split Games. Further, we uncover evidence of a gender-specific discrimination effect in the Trust Game: when the decision to trust is motivated by strategic beliefs about one's interaction partner, our participants are significantly less likely to trust immigrant men. In contrast, our results indicate that immigrant men are not especially penalized in non-strategic interactions. In sum, our mixed results highlight the importance of conceptually differentiating different interaction contexts when understanding the conditions under which (gendered) discrimination occurs.

## 2. Related Literature

### 2.1. Discrimination in Intergroup Relations

Discrimination in intergroup relations is commonly approached from the perspective of Social Identity Theory, which argues that humans have a psychological disposition for social categorization and differentiation between in-group and out-group members (Tajfel et al., 1979; Yamagishi and Mifune, 2008; Balliet et al., 2014). The main finding of this literature is that individuals have a tendency to display in-group bias, and are thus more likely to extend pro-social behavior to members of their own group rather than to out-group members.

This literature has convincingly documented how ingroup favoritism can emerge even in minimal group experiments with arbitrary distinctions between groups (e.g., preferences over paintings) (Oakes and Turner, 1980). We may therefore expect to see similar patterns in real-life situations. Recent applications of Social Identity Theory have posited that ethnic differences constitute salient group boundaries in contemporary European societies (Winter and Zhang, 2018; Bourabain and Verhaeghe, 2019; Choi et al., 2019; Zhang et al., 2019). As such, we expect that:

*(H1) Host population members are more pro-social toward in-group members than toward out-group members (individuals of immigrant origin)*.

### 2.2. Gender and Discrimination

Although Social Identity Theory provides a plausible mechanism linking ethnic differences to discrimination, it offers only limited insight on the interplay between ethnicity and gender. For that, we must turn to two additional theoretical perspectives. First, building from evolutionary theory, Navarrete et al. (2010) have developed an “outgroup-male-target” hypothesis suggesting that the basis for discrimination and prejudice stems from outgroup threats in intergroup conflict. Since this conflict has been largely perpetuated by male aggressors in human evolutionary history, these authors argue that discrimination will be targeted specifically against outgroup men.

Furthermore, another approach leading to the same expectation—that of more negative out-group bias toward young immigrant men—points to the argument that young men are more likely to commit certain hideous and violent crimes (or are at least more likely to be perceived as doing so), and are thus feared more (Gambetta and Hertog, 2017; Ward, 2019).

This line of research is closely related to social-psychological work on the Integrated Threat Theory, which has linked negative attitudes and prejudice toward immigrants to different types of threat, including realistic and symbolic threat (Stephan and Stephan, 2018)^1^. Even if immigrants do not commit crimes at a higher rate than natives (Feltes et al., 2018), adding more people of this potentially “risky” demographic may *per se* be a reason for natives to perceive immigrant men as a greater security threat.

Lastly, research has also shown that immigrant groups with many young men from Muslim-majority countries are significantly more likely to be perceived as a cultural threat (Ward, 2019). This aligns with cross-country survey evidence (e.g., the World Value Survey) that suggests that cultural values in Muslim-majority countries are on average more distant from Western European societies than those in other common regions of origin (e.g., Eastern Europe) (Inglehart and Welzel, 2005). Furthermore, scholarship on immigrant assimilation has also shown that sociocultural variables such as conservative gender and family values among immigrants from Muslim-majority countries are an alternative source of unexplained ethnic group differences in terms of integration outcomes among immigrants in Europe (Koopmans, 2016). At the same time, discrimination based on the widespread belief among natives that these gender norms are incompatible with liberal ideas of gender equality has added to the marginalization of even second-generation Muslim immigrants and especially of young Muslim men (Adida et al., 2016; Drouhot and Nee, 2019), who are often perceived as instrumental in upholding these values (Higgins, 2015).

Applying these perspectives to our context, we define *gender-specific discrimination* as referring to the phenomenon that immigrant men tend to receive worse treatment than native men, while immigrant women are treated the same as native women. We expect that:

*(H2) Host population members discriminate more against immigrant men compared to native men than against immigrant women compared to native women*.

### 2.3. Discrimination in Behavioral Games

In order to study discrimination, the literature has increasingly turned to experimental designs. In particular, behavioral games have been developed to study universal patterns of human behavior and identify and compare mechanisms, such as altruism, trust and cooperation across individuals or groups (Berg et al., 1995). These have also been used “in the field” (Bouckaert and Dhaene, 2004; Abascal, 2015; Baldassarri and Abascal, 2017; Schaub, 2017).

The controlled experimental setting allows scholars to manipulate aspects of the game, such as the identity of the players involved, allowing researchers to obtain behavioral measures of ingroup bias (Schaub et al., 2020a). For example, Adida et al. (2016) used behavioral games with French natives and Muslim and Christian immigrants from Senegal to examine religious discrimination in France. They found that natives showed lower unconditional altruism (but not lower trust) toward Senegalese Muslims in comparison to Senegalese Christians. Along the same lines, Cettolin and Suetens (2019) employed a Trust Game (described below) with a representative sample of the Dutch population, and find that Dutch natives behave more opportunistically toward non-natives.

While our article is closely related to these previous studies, our primary contribution is to focus attention on the intersection of gender and ethnicity in evaluating discrimination effects. Further, we aim to understand how patterns of gendered discrimination vary across different game contexts. Previous research has documented discrimination in some games but not others (Bouckaert and Dhaene, 2004; Adida et al., 2016; Cettolin and Suetens, 2019; Baldassarri et al., 2020), suggesting that a closer examination of the mechanisms underlying discrimination is warranted.

## 3. Methods

### 3.1. Research Setting and Sample

To evaluate these hypotheses, we turn to behavioral games embedded within a large-scale survey that we fielded between March and June 2018. The survey was implemented by the CATI Lab at the University of Jena^2^. Our data comprises a random sample of 1,243 native Germans from more than 200 rural municipalities or small towns in all five states of the former German Democratic Republic (GDR) who agreed to participate in an incentivized online-survey on the topic of “Community and Society in Germany.”^3^ For more details on our sampling strategy, see Schaub et al. (2020b)^4^.

Our study is situated in a context that is fairly common, politically important, but rarely studied: the rural hinterlands of a country, where the presence of foreigners is low, but anti-immigrant sentiments are widespread. In such areas, right-wing populists have continuously increased their vote shares, often mobilizing their voters with an anti-immigrant platform (Alba and Foner, 2017). Our study area is no exception to this trend: between the two general elections (2013 and 2017) that bracketed the onset of the “refugee crisis,” support for Germany's populist right-wing party *Alternative für Deutschland* (AfD) surged from 6 to 25%.

Participants were first recruited by phone based on a random sample of telephone numbers using a protocol that allowed targeting specific zip-code areas. During the call, participants were invited to answer an online survey and to take part in the behavioral games on a website that we programmed for this purpose using oTree (Chen et al., 2016). The survey took an average of 30 min, and participants received a variable compensation of 10–20 Euros (2–4 times the federal hourly minimum wage), with the exact amount depending on their decisions in the behavioral games.

Overall, 47% of the sample was male, and the median age was 53 years (which is slightly older than the East German average of around 46 years). Forty-three percent of the sample had a university-qualifying high school education (*Abitur*). In terms of political alignment, 11% reported having voted for the AfD in the 2017 National Elections. This number is lower than the regional average of 25%, indicating that our sample appears to be less xenophobic than the typical voter in the area. Nonetheless, right-wing attitudes were also common in the sample, with 38% of our respondents supporting the statement that “foreigners only come to exploit the welfare system,” and 36% endorsing the position that “child support should only be paid to Germans.” Table 1 presents a full description of sample demographics and the summary statistics for the different behavioral games.

**Table 1:** Summary statistics.

**Variable**	**Mean**	**Std. Dev**.	**Min**.	**Max**.	**N**
*Behavioral Game Outcomes*					
Amount Sent in Dictator Game	2.398	1.579	0	5	2,486
Amount Sent by Ego in Trust Game	2.800	1.592	0	5	2,486
Difference in Split Game: Amount Given to	−0.293	1.545	−5	5	1,243
Minority Alter Minus Amount Given to Native Alter					
Age	52.549	13.985	18	88	1,243
Male	0.47	0.499	0	1	1,243
*Education*					
Abitur	0.427	0.495	0	1	1,243
Realschule or Fachhochschulreife	0.526	0.500	0	1	1,243
Hauptschule or lower	0.047	0.211	0	1	1,243
*Employment Status*					
Full-time employed	0.498	0.500	0	1	1,231
Part-time employed	0.180	0.384	0	1	1,231
Other status	0.323	0.468	0	1	1,231
*Party voted for in 2017 national election*					
CDU	0.256	0.436	0	1	1,150
SPD	0.155	0.362	0	1	1,150
LINKE	0.211	0.408	0	1	1,150
AfD	0.122	0.327	0	1	1,150
GREENS	0.082	0.274	0	1	1,150
*Rightwing Attitudes*					
Foreigners only exploit welfare state	3.798	1.768	1	7	1,122
Only Germans should receive child support	3.648	2.193	1	7	1,098

### 3.2. Description of Behavioral Games

To evaluate our hypotheses, we employed a variety of behavioral games. Survey respondents first participated in a Dictator Game (DG) in which they were provided with an endowment of 5 EUR and asked to decide how much of this money they wanted to give to another individual (Alter). Participants kept the difference between the endowment and the amount of money they gave to Alter. Since an individual's payoff in this game depends upon his/her choice alone, the DG has been commonly understood to capture pro-social behavior motivated by non-strategic considerations such as altruism or inequality aversion (Camerer, 2011).

**Figure 1:**
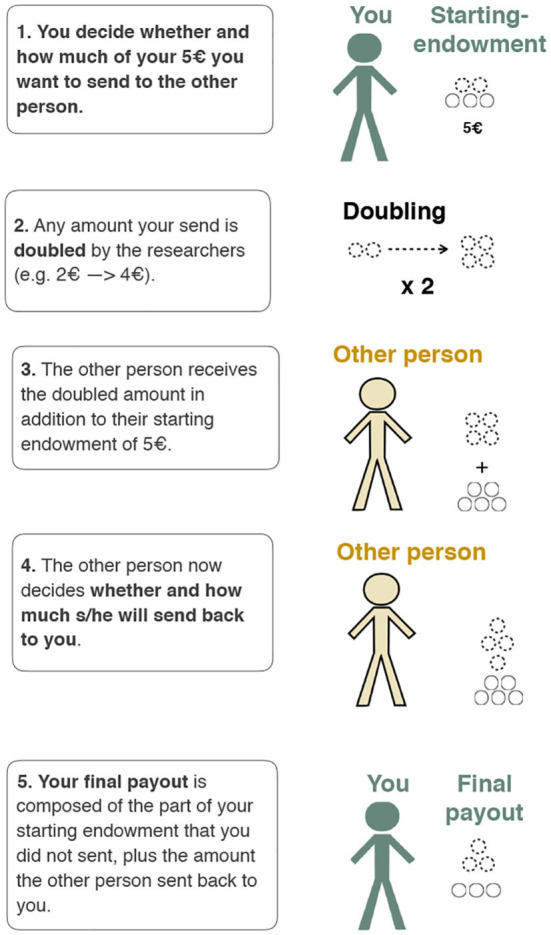
Screenshot of the Trust Game explanation.

Second, respondents took part in a Trust Game (TG) (Berg et al., 1995; Glaeser et al., 2000; Gereke et al., 2018). This game is similar to the DG in that participants also received an endowment of 5 EUR which they could share with an Alter. However, every EUR the participant chooses to share is multiplied (in our case doubled) by the researchers before being passed to Alter. Finally, Alter then decides how much of this money should be *passed back* to the participant. Since the money is doubled in the game, it is possible for the participant to gain more then s/he possessed initially – but only if Alter returns more than what was sent. The trust game therefore captures the features highlighted in Coleman (1994)'s definition of trust, namely that (1) the decision of trust is voluntary; (2) the decisions of the truster and Alter (the trustee) are sequential; (3) only if the truster shows trust can the trustee decide to abuse the demonstrated trust; (4) the truster becomes vulnerable to exploitation by showing trust. Figure 1 presents a screenshot of how the game was explained to respondents.

The Trust Game differs importantly from the DG in that it introduces an element of strategic calculation: if the participant believes that Alter is trustworthy (i.e., that he/she will pass some of the money back), it makes sense to send money in the first place as this increases the size of the pie to be shared. However, if the participant believes that Alter is untrustworthy (i.e., that he/she will keep all of the money), then it is rational to send nothing. We measure discrimination by comparing the amounts of money that immigrant Alters are sent compared to native Alters, and we can evaluate gender- specific discrimination by comparing the results for immigrant female Alters compared to native female Alters and immigrant male Alters compared to native male Alters.

While the DG and TG are commonly employed to capture elements of two-party strategic and non-strategic behavior, we also implemented a Split Game (SG) to capture situations where the participant must decide how to allocate resources (10 EUR) between two different Alters (Peterson, 2016). This game differs from the DG and TG in that the participant's own payoff is unaffected by his/her decision. This is an important difference because it eliminates self-regarding preferences or egoism, and thus provides a measure of discrimination that is less likely to be confounded or biased by other mechanisms. Moreover, the SG allows for direct comparison between in- and out-group members, while the DG and TG provide information regarding preferences for only one Alter in each round. Importantly, the direct comparison of two individuals in the SG also mirrors more closely a range of situations where decision-makers have to make a choice between two individuals (i.e., between two job candidates who compete for one position).

### 3.3. Treatment Manipulations

**Figure 2:**
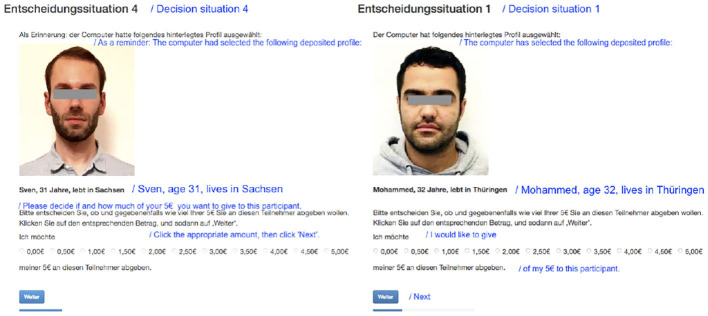
Screenshot example of German and immigrant profiles in the Dictator Games.

Participants were randomly presented with information about different Alters on which they could condition their decision in each of the games. Each Alter profile contained a picture, as well as basic information such as Alter's name, age (between 29 and 34 years) and federal state of residence (see Figure 2). By providing pictures and using stereotypical German vs. Middle Eastern names, we could manipulate perceptions of Alter's gender and immigrant origin. Overall, we drew upon a set of profiles from eight individual Alters that were carefully selected on gender, age, ethnicity, and physical appearance. This selection was based on the results from a pre-test with a total of 37 pictures on Amazon's Mechanical Turk (MTurk) where we had the photos rated in terms of perceived expressed emotions (happy, angry, fearful, sad, neutral) and attractiveness. These are factors known to influence facial cues for trustworthiness and cooperative decisions (Todorov et al., 2015). Our final sample consisted of four ethnic Germans (two male and two female) and four individuals with migration background (again two male and two female) who were closely matched across all perceived traits^5^^,^^6^.

Participants played two rounds of both the DG and the TG: once with a German Alter and once with an immigrant Alter. We randomized the order in which profiles were shown (i.e., 50% of participants encountered an immigrant Alter first, while the remainder encountered a German Alter first) in order to account for potential order effects (e.g., participants might become, in general, less altruistic over time). We also randomized the Alters' gender across the two profiles. This design allows us to measure (gendered) discrimination by estimating how behavior in the DG and TG changes as a response to the interaction of Alters' gender and ethnicity. Further, because we employ a within-subjects design, we can include respondent-level fixed effects in our models.

Randomization of profiles in the Split Game (SG) proceeded somewhat differently: here participants were shown two profiles side-by-side, one of a (randomly-chosen) German Alter and the other depicting a (randomly-chosen) immigrant Alter. The left-right placement of German vs. immigrant profiles on the screen was also randomized. However, we constrained the randomization such that both Alters had to be of the same gender. Since the two profiles are shown together, participants played the SG only once, and we measure discrimination as deviation from an even 50-50 split in favor of the German Alter.

## 4. Results

Results for all three games were analyzed using linear regression models estimated by OLS. For models involving the DG and TG, standard errors are clustered at the individual level because respondents made two decisions each. Huber-White robust standard errors are used in SG models^7^.

**Figure 3:**
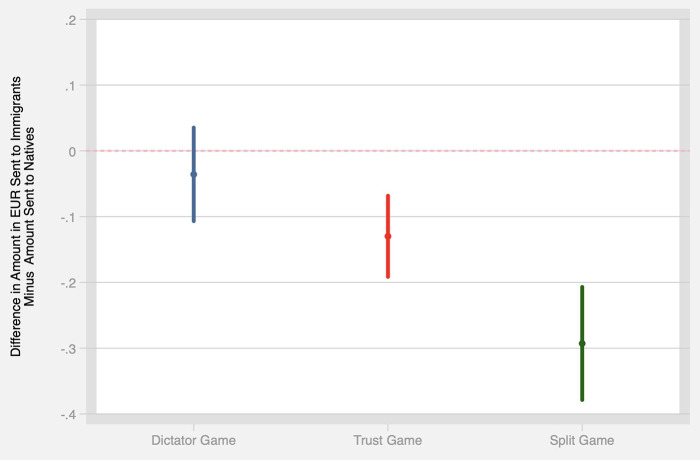
The difference in the amount of money (in EUR) shared with immigrants compared to natives in three behavioral games: the Dictator Game, the Trust Game, and the Split Game. Error bars indicate 95% confidence intervals.

To test H1, we first compare the amount sent to native vs. immigrant Alters in the DG and TG. Results are presented graphically in Figure 3, while statistical tests with respondent fixed-effects to account for our within-subjects design are reported in Model 1 of Tables 2, 3. Native Alters in the DG received 2.42 EUR on average, while immigrant Alters received 2.38 EUR. This difference is substantively small, and not statistically significant at conventional levels. In contrast, immigrant Alters did receive significantly smaller offers in the TG (2.87 EUR for native Alters compared to 2.74 EUR for immigrant Alters), and the effect is statistically significant at *p* < 0.001. Both DG and TG results remain robust after controlling for the order in which Alters' pictures were displayed (see Model 2 in Tables 2, 3).

**Table 2:** Amount of money (in EUR) sent to Alter in the Dictator Games.

	**(1)**	**(2)**	**(3)**	**(4)**	**(5)**
Immigrant Alter	−0.036	−0.029	−0.033	0.037	0.050
	(0.036)	(0.036)	(0.036)	(0.062)	(0.061)
Male Alter			−0.196^***^	−0.127^+^	−0.119^+^
			(0.051)	(0.072)	(0.071)
Immigrant × Male				−0.139	−0.151
				(0.103)	(0.102)
Second Decision		0.199^***^			0.200^***^
		(0.036)			(0.036)
Constant	2.416^***^	2.313^***^	2.513^***^	2.478^***^	2.371^***^
	(0.018)	(0.027)	(0.029)	(0.038)	(0.041)
**N**	2,486	2,486	2,486	2,486	2,486
Respondent Fixed Effects	Yes	Yes	Yes	Yes	Yes

Notes: Standard errors are reported in parentheses (

+ *p < 0.10*,

^*^p < 0.05,

^**^p < 0.01,

*** *p < 0.001)*.

**Table 3:** Amount of Money (in EUR) sent to Alter in the Trust Games.

	**(1)**	**(2)**	**(3)**	**(4)**	**(5)**
Immigrant Alter	-0.130^***^	-0.136^***^	-0.126^***^	0.001	-0.011
	(0.031)	(0.031)	(0.031)	(0.053)	(0.052)
Male Alter			-0.218^***^	-0.091	-0.098
			(0.043)	(0.062)	(0.061)
Immigrant × Male				-0.253^**^	-0.242^**^
				(0.089)	(0.088)
Second Decision		-0.176^***^			-0.175^***^
		(0.031)			(0.031)
Constant	2.865^***^	2.956^***^	2.972^***^	2.910^***^	3.003^***^
	(0.016)	(0.024)	(0.028)	(0.035)	(0.039)
*N*	2486	2486	2486	2486	2486
Respondent Fixed Effects	Yes	Yes	Yes	Yes	Yes

Notes: Standard errors are reported in parentheses (^*^p < 0.05,

** *p < 0.01*,

*** *p < 0.001)*.

Finally, we turn to discrimination in the SG by examining the extent to which monetary distributions differ from an even split (see Figure 3). This inequality is captured in the constant term in Model 1 in Table 4. This model simply estimates the average deviation from 50-50, such that a statistically *insignificant* coefficient on the constant term indicates that splits are not systematically biased toward either natives or immigrants. In contrast, we estimate that immigrant Alters received, on average, 0.29 EUR less than their share of the even split. This estimate is statistically significant at *p* < 0.001, and is also substantively much larger than the effects we uncover in the DG and TG.

**Table 4:** Deviation from an even split between in- and out-group in the Split Game.

	**(1)**	**(2)**
Male Alters		−0.043
		(0.088)
Constant	−0.293^***^	−0.272^***^
	(0.044)	(0.064)
**N**	1,243	1,243

Notes: Standard errors are reported in parentheses (^*^p < 0.05, ^**^p < 0.01,

*** *p < 0.001)*.

Overall, we find partial evidence in favor of H1: while we detect no discrimination when it comes to pro-social behavior as captured in the DG, discrimination does appear with regards to trust (in the TG) and in whether resources should go to natives or immigrants (in the SG).

**Figure 4:**
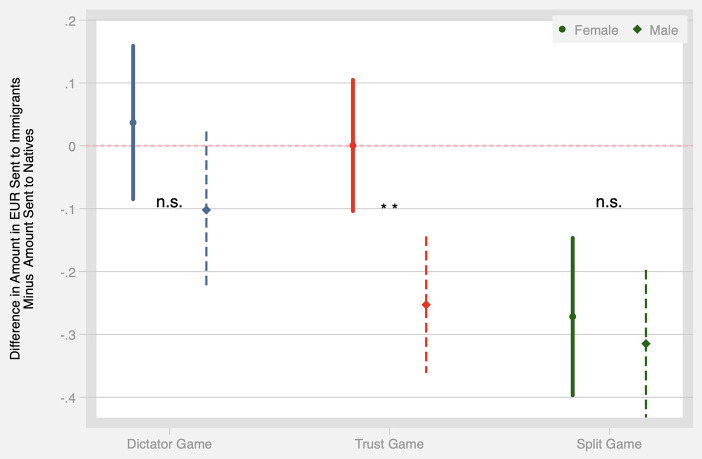
The difference in the amount of money (in EUR) shared with immigrants compared to natives for male and female Alters in three behavioral games: the Dictator Game, the Trust Game, and the Split Game. Error bars indicate 95% confidence intervals.

To test H2, we rely on the fact that respondents were randomly paired with female vs. male Alters in the DG and TG. Since the ethnicity and gender of Alters in the DG and TG were fully crossed, we include an ethnicity × gender interaction term to test whether male immigrants are the subject of greater discrimination than female immigrants. The results are presented graphically in Figure 4, while the estimated coefficients are presented in Models 3–5 of Tables 2, 3.

Turning first to the DG results, Model 3 of Table 2 shows that male Alters (of any ethnicity) receive offers around 0.20 EUR lower (*p* < 0.001). However, when interacting Alters' gender and immigrant status (Model 4), we see no additional penalty for male immigrants [the difference in margins natives and male immigrants is insignificant at *p* = 0.11 (see Table S4 with margins in Supplementary Materials)]. Again, this result is robust to controlling for order effects (Model 5).

Next we turn to results in the TG (see Models 3–5 of Table 3). Here Model 3 shows that, in addition to the main effect of discrimination against immigrant Alters discussed above, there is also a penalty for male Alters, who receive on average 0.22 EUR less than females (*p* < 0.001). However, both main effects lose significance once an interaction term is included in Model 4. In other words, it appears that the lower trust shown toward both immigrants and men is driven by reactions toward male immigrants, who are estimated to receive 0.25 EUR less than male natives^8^. As before, these results are also robust to controls for decision order (Model 5)^9^.

Finally, turning to the SG, recall that participants were randomized into seeing either (a) native female vs. immigrant female Alters or (b) native male vs. immigrant male Alters. Thus, we can examine the extent of gender specific discrimination by comparing splits in (a) vs. (b). The results are shown in Model 2 of Table 4 and presented graphically in Figure 4. The constant term in Model 2 now represents the deviation from an even split in (a), while the coefficient on male alters indicates the extent to which distributions in (b) differ from this all-female baseline. The coefficient on male Alters is substantively small and statistically insignificant, which indicates no additional gender-specific discrimination in the SG.

Overall, we find partial evidence in support of H2. In the strategic interaction environment of the Trust Game, host population members discriminate against immigrant men, while immigrant women face no such discrimination. In contrast, no gender-specific penalty is observed in the non-strategic games. Neither the DG (no discrimination against either gender) nor the SG (equal discrimination against both genders) support the hypothesis that male immigrants are particularly likely to be the targets of prejudice.

## 5. Discussion

Recent migration from Muslim-majority countries has sparked discussions across Europe and the U.S. about the supposed threat posed by new immigrants (Adida et al., 2019; Valentino et al., 2019; Helbling and Traunmüller, 2020). Our study presents results from a large-scale online survey with residents of Germany's eastern regions that included several behavioral games designed to capture pro-social behavior and discrimination against immigrant men and women from Muslim-majority countries.

Our study makes three contributions to the literature on discrimination of immigrants and ethnic minorities. The first contribution concerns the study setting: Our study is set in a fairly common and politically important context where anti-immigrant sentiments are generally high but which is rarely studied. We use a unique sample of rural respondents in the five eastern German *Bundesländer*, which is more general than the samples typically employed in experimental studies on discrimination, prejudice and stereotyping. Of course, our sampling strategy may have biased us toward the identification of discrimination since anti-immigrant attitudes are generally more pronounced in non-urban areas. However, given the structural similarity of our study region to other rural areas in Germany and Europe, our findings may extend to similar settings in which there is strong anti-immigrant sentiment. However, it remains to be tested in future work whether our findings replicate and generalize beyond this context and population.

Second, we highlight and systematically examine the role of gendered ethnic discrimination against immigrants and ethnic minorities, which has so far received little attention among social scientists. Our results complement those of Ward (2019) who finds low levels of support for groups of young male refugees in Germany, which he argues is driven by perceptions of young men as cultural and security threats. In Germany, many of the recent refugee arrivals were young and male, which has resulted in a growing imbalance in the gender ratio and increased the competition for female partners. This competitive situation in the mating market has been identified as a critical factor driving hate crimes against refugees (Dancygier et al., 2020) and highlights the importance of studying gender dynamics in immigrant discrimination research. However, one limitation of our study is that by design (since all our Alters are from Muslim-majority cultures) we are unable to disentangle between an outgroup bias against Muslim and non-Muslim immigrant men. Future research should therefore test the boundaries of this outgroup bias.

Finally, our study adds to a handful of articles using behavioral games to measure ingroup favoritism and study discrimination against immigrants and ethnic minorities in European societies (Adida et al., 2016; Cettolin and Suetens, 2019). Behavioral games are useful in the study of discrimination because they allow us to tease apart mechanisms. We focus on strategic and non-strategic pro-social behavior with respect to gendered ethnic discrimination. We find mixed evidence for gendered ethnic discrimination in this paper. While we find that natives are similarly pro-social toward men and women of immigrant origin in non-strategic situations, we also observe that natives are significantly less likely to trust immigrant men in strategic encounters. Importantly, our results suggest that gendered ethnic discrimination is not simply taste-based (Becker, 1957) since in this case we should observe discrimination in the non-strategic DG and SG. Instead, gendered ethnic discrimination appears to be driven by an unwillingness among natives to trust male immigrants. However, a limitation of the current study that future research should address is to better understand how our treatment effects are moderated by personality factors, such as authoritarianism (Adorno et al., 1950) or implicit biases (Rudman and Ashmore, 2007), which may differ across individual respondents. The estimation of such heterogeneous treatment effects may shed light on the social-psychological mechanisms underlying our results.

Overall, our results call for a better understanding of the conditions under which discrimination occurs with respect to pro-social behaviors and the need to develop a theory for understanding immigrant discrimination that takes into account the differences in reception faced by male and female immigrants.

## Data Availability Statement

The dataset generated for this study can be found in the Havard Dataverse Project Gereke, Johanna, 2020, “Replication Data for Gendered discrimination against immigrants,” https://doi.org/10.7910/DVN/G0W9MH, Harvard Dataverse, V1, UNF:6:xGkgR2Q+V62Qd+0mulJdoQ== [fileUNF].

## Ethics Statement

Written informed consent was obtained from the individuals for the publication of any potentially identifiable images or data included in this article.

## Author Contributions

JG, MS, and DB designed research. MS and JG performed research and analyzed data. JG wrote the first draft of the manuscript. MS wrote sections of the manuscript. All authors contributed to manuscript revision, read and approved the submitted version.

## Conflict of Interest

The authors declare that the research was conducted in the absence of any commercial or financial relationships that could be construed as a potential conflict of interest.

## Footnotes

^1^We understand prejudice here as biased beliefs, thoughts, and attitudes about a group of people. Discrimination is defined as differential behavior toward a specific group of people based on their ethnicity and gender.

^2^Prior to data collection, the study received ethical approval from the Ethics committee of the grant hosting institution (Bocconi University).

^3^The average population of the municipalities in our sample is 3,116 with an average population density of 59 persons/km^2^. We excluded 46 individuals with a migration background and 31 individuals that were subject to a randomization error in terms of the profiles shown from our analyses. The results presented below are robust to using the full sample.

^4^The respondents were from closely-matched municipalities, half of which received refugees in the wake of the 2015 refugee crisis. Our starting population were all municipalities in the Eastern German *Bundesländer* of *Mecklenburg-Vorpommern, Sachsen-Anhalt, Brandenburg, Thüringen* and *Sachsen*. A matching strategy at the municipality level (excluding municipalities with foreigner shares above 1.5% and less than 6.6 km distance between them) allowed us to study how local exposure to refugees affects right-wing support, which we report in Schaub et al. (2020b). We find that refugee allocation has no effect on immigrant attitudes and voting behavior. With regards to our analyses here, we find similar treatment effects in the behavioral games for people living in municipalities with and without refugees (results not shown).

^5^For the two immigrant women, we included pictures both with and without a Muslim headscarf (hijab), since approximately 28% of Muslim women in Germany report wearing a headscarf (Haug et al., 2009). Overall, we did not find any significant penalty for immigrant women wearing headscarves and therefore pool the immigrant women profiles in our analyses.

^6^The pre-test participants whose pictures we used as “Alter” in the behavioral games were paid the average amounts handed to them by the survey respondents after the completion of the survey.

^7^We additionally present all regression results with standardized regression coefficients in Tables S1–S3 in the Supplementary Materials.

^8^They also receive 0.35 EUR less than native women, and 0.34 EUR less than immigrant women. See margins in Table S5 in the Supplementary Material.

^9^We also explore a specific variant of H2, namely that there could be gender-specific differences in gender-discrimination. To that effect, we estimate a three-way interaction effect with respondent's gender, gender of alter and immigrant status for the trust game, which is the only game in which we find gendered discrimination against immigrant alters. Briefly, we find no significant differences in discrimination against male immigrants between female and male respondents. Please refer to Tables S6 and S7 and Figure S1 in the Supplementary Materials for more information.
