# Supplementary figures and images for: Gendered Discrimination Against Immigrants: Experimental Evidence
Source: Front Sociol. 2020 Sep 3;5:59. doi: 10.3389/fsoc.2020.00059 (PMC8022493; doi:10.3389/fsoc.2020.00059)

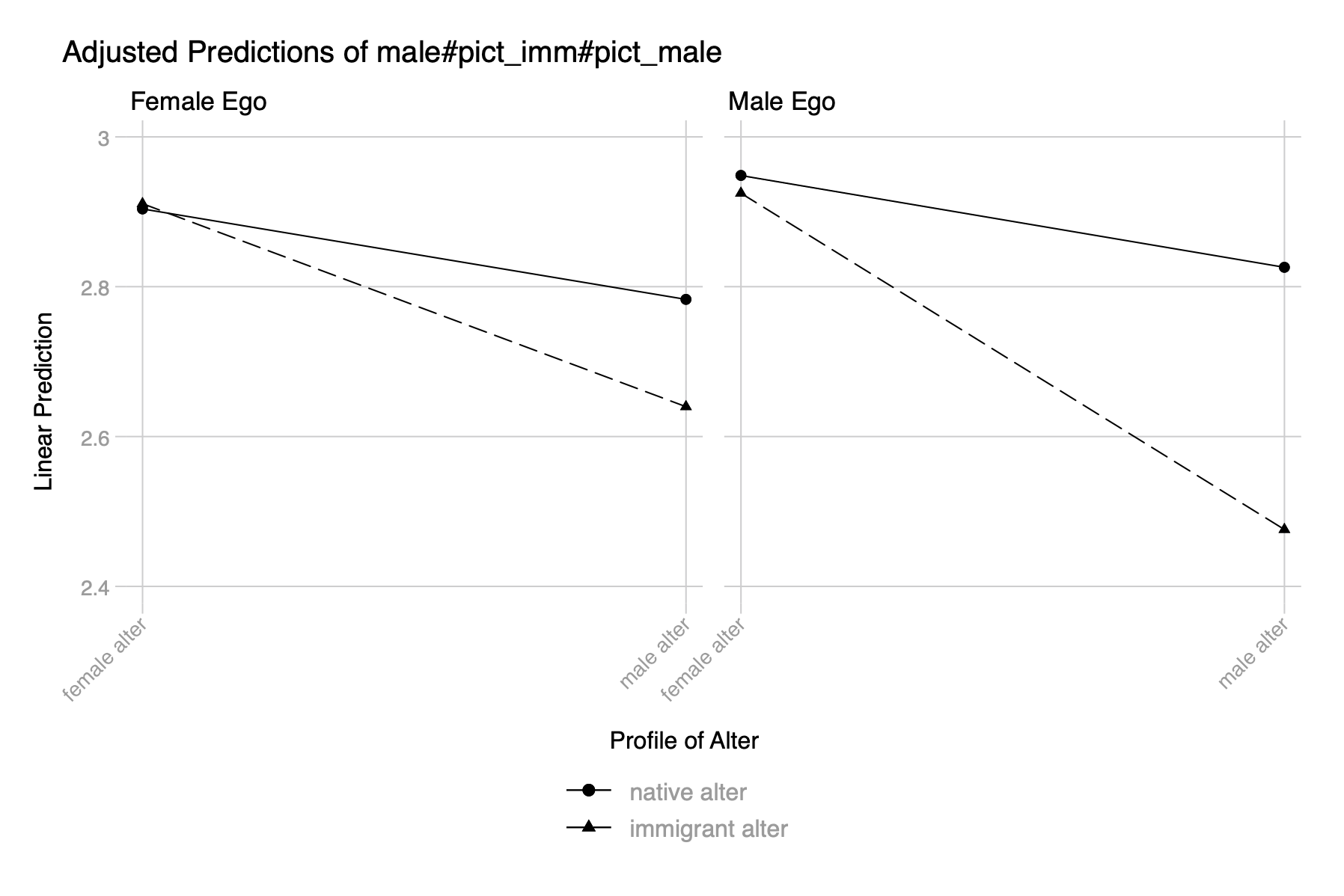

Supplement: Supplementary file 1 [file Image_1.PNG]
