# Supplementary material for: Gendered Discrimination Against Immigrants: Experimental Evidence
Source: Front Sociol. 2020 Sep 3;5:59. doi: 10.3389/fsoc.2020.00059 (PMC8022493; doi:10.3389/fsoc.2020.00059)
Supplement: Supplementary file 2 [file Data_Sheet_1.ZIP › SM/SM_Frontiers.pdf]

Supplementary Material

1 SUPPLEMENTARY TABLES

1.1 Results with Standardized DVs

Table S1. Amount of Money (in EUR) sent to Alter in the Dictator Games (Standardized Coefficients)

	(1)	(2)	(3)	(4)	(5)
Immigrant Alter	-0.023	-0.018	-0.021	0.023	0.032
	(0.023)	(0.023)	(0.023)	(0.039)	(0.039)
Male Alter			-0.124***	-0.080+	-0.076+
			(0.032)	(0.046)	(0.045)
Immigrant × Male				-0.088	-0.096
				(0.065)	(0.064)
Second Decision		0.126***			0.126***
		(0.023)			(0.023)
Constant	0.011	-0.054**	0.073***	0.051*	-0.017
	(0.011)	(0.017)	(0.018)	(0.024)	(0.026)
N	2486	2486	2486	2486	2486
Respondent Fixed Effects	Yes	Yes	Yes	Yes	Yes

Notes: Standard errors are reported in parentheses ($^{+}$ $p < 0.10$, * $p < 0.05$, ** $p < 0.01$, *** $p < 0.001$).

Table S2. Amount of Money (in EUR) sent to Alter in the Trust Games (Standardized coeffcients)

	(1)	(2)	(3)	(4)	(5)
Immigrant Alter	-0.082***	-0.086***	-0.079***	0.000	-0.007
	(0.020)	(0.020)	(0.020)	(0.033)	(0.033)
Male Alter			-0.137***	-0.057	-0.061
			(0.027)	(0.039)	(0.039)
Immigrant × Male				-0.159**	-0.152**
				(0.056)	(0.055)
Second Decision		-0.110***			-0.110***
		(0.020)			(0.019)
Constant	0.041***	0.098***	0.108***	0.069**	0.128***
	(0.010)	(0.015)	(0.017)	(0.022)	(0.024)
N	2486	2486	2486	2486	2486
Respondent Fixed Effects	Yes	Yes	Yes	Yes	Yes

Notes: Standard errors are reported in parentheses (* $p < 0.05$, ** $p < 0.01$, *** $p < 0.001$).

Table S3. Amount of Money (in EUR) split between in- and out-group in the Split Game (Standardized coeffcients)

	(1)	(2)
Male Alters		-0.028
		(0.057)
Constant	-0.000	0.014
	(0.028)	(0.041)
N	1243	1243

Notes: Standard errors are reported in parentheses (* $p < 0.05$, ** $p < 0.01$, *** $p < 0.001$).

1.2 Average Marginal Effects of Components of Interaction Terms in Dictator Game Trust Games

Table S4. Marginal Effects Estimates for Interaction Terms in the Regression Analysis of the Dictator Game Reported in Model 5 of Table 2 in Main Manuscript

	(1)
Native#Female Alter	2.471<br>(0.038)
NativeImmigrant Alter	2.352<br>(0.042)
Immigrant#Female Alter	2.522<br>(0.041)
Immigrant#Male Alter	2.251<br>(0.038)
N	2486

Notes: Standard errors are reported in parentheses

Table S5. Marginal Effects Estimates for Interaction Terms in the Regression Analysis of the Trust Game Reported in Model 5 of Table 3 in Main Manuscript

	(1)
Native#Female Alter	2.916<br>(0.034)
Native#Male Alter	2.818<br>(0.034)
Immigrant#Female Alter	2.905<br>(0.033)
Immigrant#Male Alter	2.565<br>(0.035)
N	2486

Notes: Standard errors are reported in parentheses

1.3 Three Way Interactions of Respondent's Gender, Alter's Gender and Immigrant Identity in the Trust Games

In the main manuscript, we report that we only find gendered-discrimination towards immigrant men in the Trust Game but not in the DG or SG. To examine gender differences in gender-specific discrimination

in the TG, we again run linear regression models using OLS and present estimates with person fixed effects because every respondent provided decisions in two trust games (once with an in-group and once with an out-group member) in Table S6 as well as Huber-White robust standard errors clustering at the respondent level in Table S7.

Model 1 in Table S6 is equivalent to Model 4 in Table 3 in the main manuscript and shows that the interaction term of seeing a male and Immigrant Alter is significant ($p < 0.01$). This means that immigrant men receive significantly lower trust (0.25 EUR less) than any other group. In Model 2, we add the interaction for Male Ego and Immigrant Alter and find that immigrant profiles are significantly trusted less, and that male survey respondents are more distrusting of immigrant men than female survey respondents ($p < 0.05$). In Model 3, we examine the interaction between Male Ego and Male Alter and find that here too, there is a statistically significant interaction effect ($p < 0.10$). This implies that switching from female to male survey respondents is associated with a more negative effect on trust when shown a Male Alter then when shown a Female Alter.[1]

We show the three-way interaction between Immigrant Alter, Male Alter and Male Ego in Models 4 both using fixed effects (Table S6) and also using robust standard errors (Table S7). We find that although the coefficient is relatively large (β=0.15 in Table S6 and β=0.18 in Table S7), it is not statistically significant. This means that the interaction among Male Alter and Immigrant Alter is not significantly different across Female and Male Egos (survey respondents) (see also Figure S1).

[1] However, in Model 3 of Table S7, we find that this term is not significant using robust standard errors.

Table S6. Amount of Money (in EUR) sent to Alter in the Trust Games using OLS with Fixed Effects

	(1)	(2)	(3)	(4)
Immigrant Alter	0.001	-0.066^{+}		0.019
	(0.053)	(0.040)		(0.069)
Male Alter	-0.091		-0.150**	-0.063
	(0.062)		(0.056)	(0.079)
Immigrant × Male Alter	-0.253**			-0.174
	(0.089)			(0.111)
Male Ego × Immigrant Alter		-0.136*		-0.044
		(0.064)		(0.107)
Male Ego × Male Alter			-0.163^{+}	-0.074
			(0.088)	(0.125)
Immigrant Alter × Male Alter × Male Ego				-0.148
				(0.178)
Constant	2.910***	2.865***	2.914***	2.913***
	(0.035)	(0.016)	(0.022)	(0.035)
N	2486	2486	2486	2486
Respondent Fixed Effects	Yes	Yes	Yes	Yes

Notes: Standard errors are reported in parentheses ($^{+}$ $p < 0.10$, * $p < 0.05$, ** $p < 0.01$, *** $p < 0.001$).

Table S7. Amount of Money (in EUR) sent to Alter in the Trust Games using OLS with Random Effects

	(1)	(2)	(3)	(4)
Immigrant Alter	-0.005	-0.066^{+}		0.007
	(0.050)	(0.040)		(0.066)
Male Ego		0.043	0.032	0.045
		(0.089)	(0.092)	(0.105)
Male Alter	-0.117*		-0.196***	-0.121^{+}
	(0.057)		(0.053)	(0.073)
Immigrant × Male Alter	-0.242**			-0.150
	(0.083)			(0.104)
Male Ego × Immigrant Alter		-0.136*		-0.031
		(0.064)		(0.102)
Male Ego × Male Alter			-0.102	-0.002
			(0.083)	(0.114)
Immigrant Alter × Male Alter × Male Ego				-0.176
				(0.167)
Constant	2.923***	2.844***	2.908***	2.904***
	(0.052)	(0.057)	(0.059)	(0.068)
N	2486	2486	2486	2486

Notes: Standard errors are reported in parentheses ($^{+}$ $p < 0.10$, * $p < 0.05$, ** $p < 0.01$, *** $p < 0.001$).

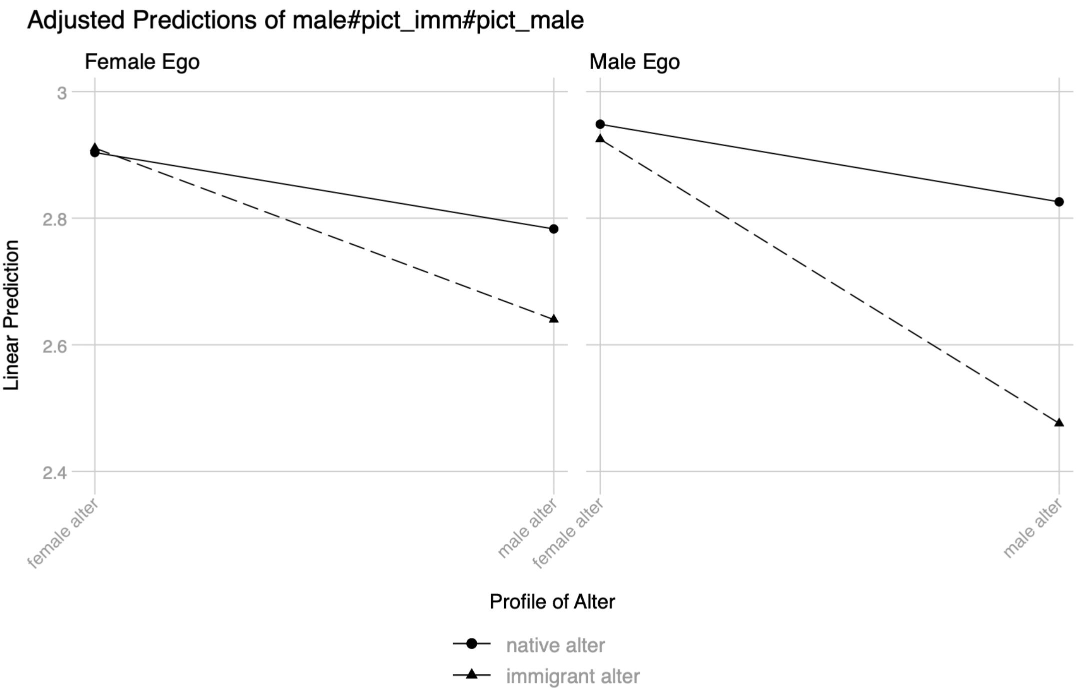

Figure S1. Margins of Three Way Interaction Estimates in the Trust Game (Table S7): Gender Ego, Immigrant vs. Native Alter, Gender Alter
